# The effect of opioid-sparing anesthesia regimen on short-term cognitive function after thoracoscopic surgery: a prospective cohort study

**DOI:** 10.1186/s13741-022-00278-9

**Published:** 2022-08-16

**Authors:** Hong Zhao, Qiaoyu Han, Chuan Shi, Yi Feng

**Affiliations:** 1 Department of Anesthesiology, Peking University People’s Hospital, Beijing, 100044 China; 2 Peking University Sixth Hospital, Peking University Institute of Mental Health, National Clinical Research Center for Mental Disorders (Peking University Sixth Hospital), NHC Key Laboratory of Mental Health (Peking University), Beijing, 100191 China

**Keywords:** Thoracoscopic surgery, Postoperative cognition, Cogstate computerized battery, Prospective cohort study, Erector spinae plane block

## Abstract

**Background:**

As type of surgery and opioids are suggested risk factors for the development of cognitive decline after surgery, we evaluated the effect of an opioid-sparing anesthesia regimen involving preoperative erector spinae block and continuous infusion of flurbiprofen on the incidence of cognitive decline after video-assisted thoracoscopic surgery.

**Methods:**

In this observational study, patients over 18 years old presenting for elective video-assisted thoracoscopic surgery were divided into two groups, the erector spinae plane block group (ESPB group, who received preoperative single shot of bi-level ESPB at T4 and T6 levels) and the control group who received intercostal nerve blocks through T5 to T7 intercostal spaces along mid-axillary line after surgery. Continuous infusion of flurbiprofen (8 mg/h) and intravenous oxycodone rescue (1 mg/bolus, lockout time 10 minutes) were provided as postoperative analgesics. Cognitive function was measured one day before and 48 h after surgery with brief Cogstate computerized battery (CCB).

**Results:**

There were 60 patients included with 30 in each group. Perioperative sufentanil dose was significantly reduced in ESPB group. Nine (30%) and 15 (50%) patients had delayed neurocognitive recovery in the ESPB group and the control group respectively. Psychomotor speed and visual attention tests were the two tests that patients showed cognitive decline. The results of multivariate regression revealed that patients who were more than 53.5 years of age (OR 9.213, 95% CI 1.789, 47.437, *P* = 0.008) and low levels of education (less than 9 years of complimentary education) (OR 6.829, 95% CI 1.068, 43.677, *P* = 0.042) were independent risk factors for postoperative delayed neurocognitive recovery. For subgroup analysis, ESPB could reduce the occurrence of delayed neurocognitive recovery in patients with both risk factors (6/10 (60%) vs. 11/11 (100%), *P* = 0.004) compared to the control group.

**Conclusions:**

Middle-aged people and low levels of education are independent risk factors for delayed neurocognitive recovery after thoracoscopic surgery. ESPB has the potential to prevent cognitive decline in high-risk patients.

**Trial registration:**

ChiCTR1800014508 (www.chictr.org.cn, January 17, 2018; Hong Zhao, M.D.). URL: http://www.chictr.org.cn/showproj.aspx?proj=24778. The date of the enrolment of the first participant to the trial was January 22, 2018.

## Key point statement

*Question*: As type of surgery and opioids are suggested risk factors for the development of cognitive decline after surgery, will opioid-sparing anesthesia regimen decrease the incidence of delayed neurocognitive recovery after video-assisted thoracic surgery?

*Findings*: Over 53.5 years of age and low levels of education were independent risk factors for delayed neurocognitive recovery, but preoperative erector spinae plane block could reduce the occurrence of cognitive decline in patients with both risks.

*Meaning*: An opioid-sparing multimodal anesthesia regimen is recommended to reduce the occurrence of delayed neurocognitive recovery in patients undergoing video-assisted thoracic surgery especially those with known risk factors.

## Introduction

Postoperative cognitive decline is a frequent complication following surgery, which usually occurs immediately after surgery, affecting up to 40% of patients undergoing non-cardiac surgeries (Awada et al. 2019). Delayed neurocognitive recovery is characterized as a decrement in cognitive function including concentration, memory, or learning up to 30 days after surgery (Evered et al. 2018a). Even though subtle cognitive decline in the elderly is common in general community, and overlaps with that after anesthesia, objective assessment through neuropsychological test batteries is still required due to the following two reasons. First, patients who develop postoperative cognitive decline could be labeled as “susceptibility” or potential Alzheimer’s disease candidates (Evered et al. 2016). Second, cognitive decline also affects middle-aged patients other than elderly people (Bratzke et al. 2018).

Risk factors suggested for the development of cognitive decline after surgery and anesthesia include preoperative cognitive impairment, pain, surgical inflammatory response, duration and type of surgery, hypoxemia, age, and certain medications such as benzodiazepines and diphenhydramine (Aldecoa et al. 2017). But the exact cause of cognitive decline remains unknown. A recent study investigating risk factors for cognitive decline after total knee arthroplasty under enhanced recovery after surgery (ERAS) scheme revealed that higher opioid consumption was related with the occurrence of cognitive decline (Awada et al. 2019).

Nowadays, video-assisted thoracoscopic surgery (VATS) is gaining popularity in the treatment of lung cancer with minimal invasiveness and equivalent efficacy. Given the fact that patients undergoing VATS have fixed age, unmodifiable surgical procedure, and its related inflammation, whether opioid-sparing analgesia may convey benefit in reducing the incidence of cognitive decline is of relevance in the emphasis of perioperative medicine.

Opioid-sparing anesthesia management is often realized through nerve blocks, non-steroid anti-inflammatory drugs (NSAID)s, or other medications. Paravertebral block, erector spinae plane block, and other thoracic wall blocks are all proposed to participate in postoperative pain management after VATS (Zhao et al. 2020). ESPB is to inject local anesthetics into the space between the erector spinae muscle and transverse process, nerves penetrating through this space would be infiltrated, and some drug may diffuse into the paravertebral space.

In this observational single-blind study, we investigated the effect of opioid-sparing analgesia involving preoperative single shot of erector spinae block and continuous infusion of NSAIDs on the incidence of delayed neurocognitive recovery after VATS.

## Methods

The study was conducted at Peking University People’s Hospital in Beijing, China, and Strengthening the Reporting of Observational studies in Epidemiology guidelines were followed. The study was approved by the Institutional Review Board of Peking University People’s Hospital, Beijing, China, and written informed consent were obtained from all subjects participating in the trial. The trial was registered prior to patient enrollment, registration number for clinical trials being ChiCTR1800014508 (www.chictr.org.cn, Jan 17, 2018; Hong Zhao, M.D.). In this observational single-blind study, patients aged between 18 and 75 years who were diagnosed as lung nodules and scheduled for VATS under general anesthesia were included. Patients were excluded if they were allergic to NSAIDs and had a history of asthma, peptic ulcer disease, inflammatory bowel disease, or renal deficiency.

### Study protocol

Patients were divided into two groups, the erector spinae plane block group (ESPB group, who received preoperative single shot of bi-level paravertebral block at T4 and T6 levels) and the control group (who received intercostal nerve blocks through T5 to T7 intercostal spaces along mid-axillary line after surgery). Ultrasound-guided ESPB was performed in the preparation room. Patients who did not receive ESPB due to unavailability of preparation room received intercostal nerve blocks performed by the surgeons. The dermatome block level in two groups was tested after extubation in the postoperative care unit by a resident who was unaware of patients’ grouping. Continuous infusion of flurbiprofen (8 mg/h) and intravenous oxycodone rescue (1 mg/bolus, lockout time 10 min) were provided as postoperative analgesics.

Cognitive function was measured for all patients on the day before surgery and 48 h after surgery (on postoperative day 3) using brief Cogstate computerized battery to assess psychomotor speed, visual attention, visual memory, and working memory. Cogstate computerized battery was installed in a laptop and provided to the patients by a resident who was unknown of patients grouping.

Demographic, anesthetic, surgical data, the occurrence of postoperative complications, and the postoperative recovery of patients were collected. The changes between pre- and postoperative cognitive functions and the incidence of delayed neurocognitive recovery 48 h after surgery were analyzed and compared.

### Ultrasound guided erector spinae block

**Fig 1.**
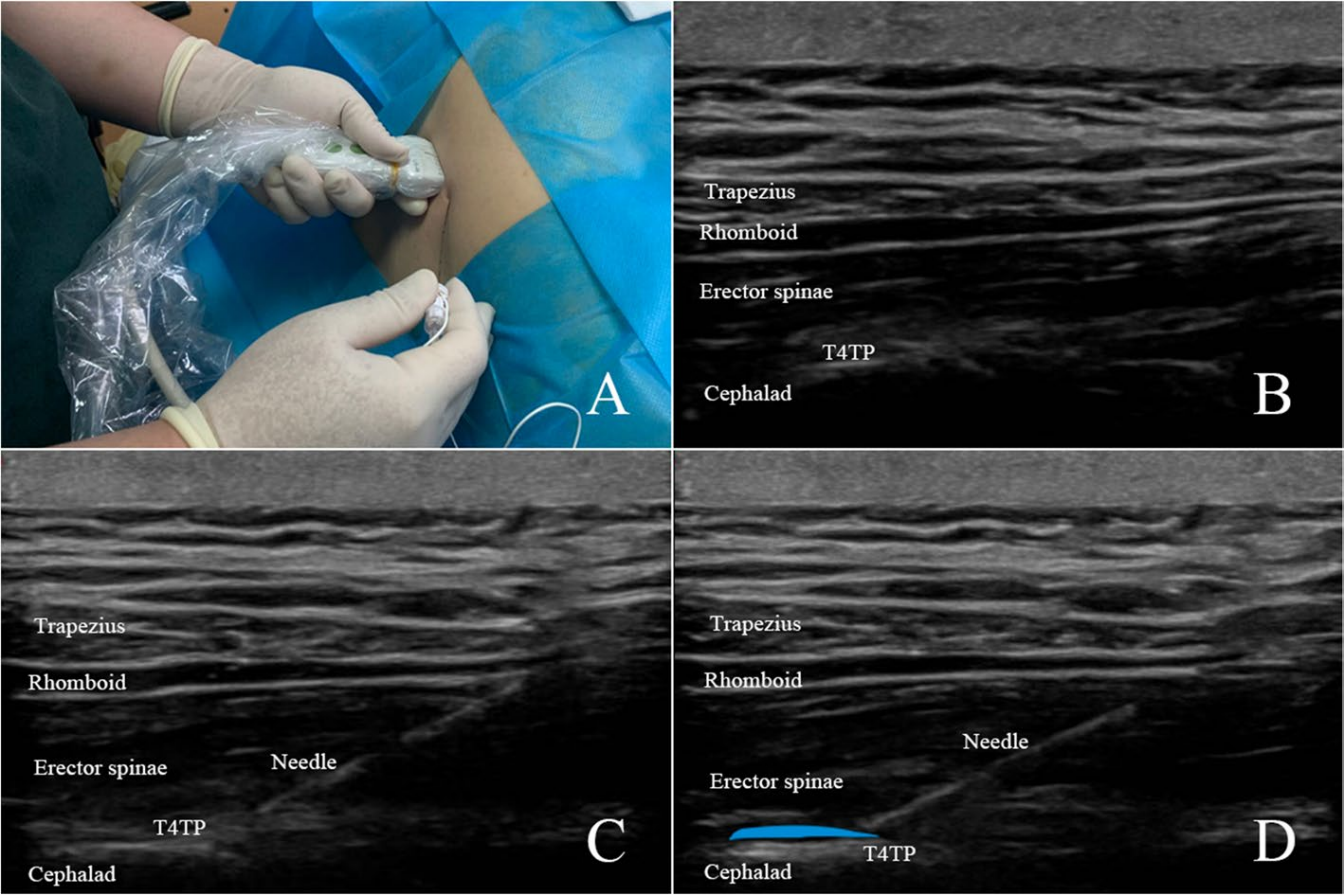
Ultrasound guided erector spinae plane block. **A** The block needle was advanced from a caudal to cranial direction. **B** Ultrasonographic image of the T4 transverse process in the middle of the image. **C** The block needle was advanced in-plane until the needle tip contacted the T4 transverse process (TP). **D** Spread of local anesthetic was observed in the plane between the erector spinae muscle and the transverse process, and erector spinae muscle lifted during the block. T4TP, transverse process

Ultrasound-guided ESPB was performed in the lateral position using longitudinal, in-plane technique under strict aseptic precautions in the preparation room. The tip of the T4 transverse process was identified using a high frequency ultrasound transducer by recognizing the fourth rib counting down from C7. The parasagittal view revealed a subcutaneous tissue, trapezius, rhomboid, and erector spinae muscle layers superficial to the transverse processes. With T4 transverse process placed in the middle of the image, a 22-gauge block needle was inserted in-plane and directed to the middle on T4 transverse process. The correct location of the needle tip in the fascial plane deep to the erector spinae muscle was confirmed by injecting 0.5 to 1 mL of saline to visualize lifting of the erector spinae muscle off the transverse process without distending the muscle. Fifteen milliliters of 0.33% ropivacaine was then injected into the erector spinae plane. Another 15 mL of 0.33% ropivacaine was injected into erector spinae plane by identifying T6 transverse process (Fig. 1).

### Intercostal nerve block

Patients in the control group received intercostal nerve blocks through T5 to T7 intercostal spaces along mid-axillary line after surgical closure performed by surgeons. One percent ropivacaine 10 mL was injected at the lower border of the fifth (3 mL local anesthetics), sixth (3 mL), and seventh rib (4 mL) along the mid-axillary line.

### Perioperative management

General anesthesia was induced with etomidate (0.3 mg/kg), sufentanil (0.3 μg/kg), and rocuronium (0.6 mg/kg), and an appropriate size of double-lumen tube was inserted. Anesthesia was maintained with continuous infusion of propofol (4–6 mg/kg·h), remifentanil (0.1–0.2μg/kg·min), and dexmedetomidine 0.4μg/kg·h to accomplish bispectral index ranging from 40 to 60, heart rate and invasive blood pressure varying from baseline ± 20%. Additional doses of sufentanil were left to the discretion of the anesthesiologist in charge to achieve adequate analgesia. Dexmedetomidine was discontinued and ondansetron 5 mg was given intravenously 30 min before the end of surgery.

A loading dose of flurbiprofen (1.5 mg/kg) was given intravenously at the end of surgery and a continuous infusion of flurbiprofen (4 mg/mL) was given at 2 mL/h with a total volume of 100 mL through a single-use infusion device (KSH**®**, KSH Medical Technology, Beijing, China). Postoperative analgesic regimen also comprised of patient-controlled intravenous oxycodone rescue (CADD**®**, Smiths Medical, Ashford, UK), set as bolus of 1 mg/2.5 mL (total volume as 40 mg/100 mL), with a lockout time of 10 min.

The total amount of intraoperative sufentanil and remifentanil were recorded. The visual rating scale (VAS) scores of pain and analgesic consumption through the patient-controlled intravenous analgesia (PCIA) pump were followed up at 24 h and 48 h after the operation. Nausea, vomiting, dizziness, and other complications were recorded.

### Cognitive function assessment

Cognitive function was measured for all patients on the day before surgery and 48 h after surgery using brief Cogstate computerized battery to assess psychomotor speed, visual attention, visual memory, and working memory. Four different tasks provided by CCB including “detection task (DET),” “identification task (IDN),” “one card learning task (OCL),” and “one-back task (OBK)” were used to assess psychomotor speed, visual attention, visual memory, and working memory respectively. The changes between pre- and postoperative cognitive functions and the presence of delayed recovery of cognitive function after surgery were analyzed. Delayed neurocognitive recovery or cognitive decline was defined as 1.96 SD decrease in 2 tests (Evered and Silbert 2018).

### Statistical analysis

The study was initially powered as a prospective cohort study. The sample size was calculated form the results of a pilot study of 10 patients who received general anesthesia with intercostal nerve blocks after surgery. Reaction time on the detection task increased from 2.65 log_10_ ms (SD = 0.11) ms before surgery to 2.85 log_10_ ms (SD = 0.15) after surgery. We assumed that preoperative ESPB would result in a smaller increase in reaction time on the detection task (approximately 50%) to a detection task speed of 2.75 log_10_ ms after surgery. Therefore, 30 patients per group were required (*α* = 0.05 two tailed, *β* = 0.2).

Statistical analysis was performed using the SPSS 24.0 statistical software package (SPSS Inc., Chicago, IL, USA). Continuous variables are expressed as mean (SD) or medians with interquartile range and categorical variables as numbers and percentages. Between-group differences were evaluated using the independent *t* test or Mann-Whitney *U* test for continuous variables and the chi-square test or Fisher exact test for categorical variables, as appropriate.

## Results

There were 60 patients included in this study with 30 patients in the ESPB group and 30 in the control group, and all patients completed the follow-up. There was no difference in demographic or surgical data (Table 1).

**Table 1:** Demographic data for patients

	Group ESPB (*n* = 30)	Group control (*n* = 30)	Statistics	*P* value
Age (years)	55 ± 11	53 ± 13		
Height (cm)	166 ± 9	168 ± 8		
Weight (kg)	72 ± 15	68 ± 14		
Male (*n* (%))	14 (46.7%)	17 (56.7%)	0.601	0.606
ASA score (*n* (%))			0.069	1.000
1	12 (40%)	13 (43.3%)		
2	18 (60%)	17 (56.7%)		
Hypertension (*n* (%))	8 (26.7%)	10 (33.3%)	0.317	0.669
Coronary heart disease (*n* (%))	6 (20%)	1 (3.3%)	4.043	0.103
Diabetes (*n* (%))	4 (13.3%)	2 (6.7%)	0.741	0.671
More than 53.3 years (*n* (%))	18 (60%)	15 (50%)	0.606	0.436
Education years < 9 (*n* (%))	10 (33.3%)	15 (50%)	1.714	0.295
Smoking (*n* (%))	10 (33.3%)	8 (26.7%)	0.317	0.779
Drinking alcohol (*n* (%))	6 (20%)	13 (43.3%)	3.774	0.095

*Group ESPB* group erector spinae plane block, *Group Control* group intercostal nerve block. Data are shown as mean ± SD, median (Q1, Q3), or numbers (%)

Intraoperative sufentanil use was significantly reduced in the ESPB group (0.43 ± 0.11 vs. 0.54 ± 0.10 μg/kg, *P* < 0.001) compared to the control group. Cumulative opioid consumption (counting in postoperative oxycodone rescue, oxycodone 1 mg = sufentanil 1ug (Han et al. 2018)) at 48 h after surgery was significantly lower in the ESPB group (0.52 ± 0.13 vs. 0.69 ± 0.22 μg/kg, *P* = 0.001) in comparison with the control group. Pain score at postoperative 48 h was not different between two groups. Chest tube indwelling time (2.5 (Evered et al. 2018a; Evered et al. 2016) vs. 2.5 (Evered et al. 2018a; Evered et al. 2016) days) and length of hospital stay (3.5 (Evered et al. 2016; Bratzke et al. 2018) vs. 4 (Evered et al. 2016; Aldecoa et al. 2017) days) were not different between two groups (Table 2).

**Table 2:** Surgical and anesthetic data

	Group ESPB (*n* = 30)	Group control (*n* = 30)	Statistics	*P* value
Left lung surgery (*n* (%))	10 (33.3%)	13 (43.3%)	0.585	0.597
Surgical procedure (*n* (%))			0.067	1.000
Segmentectomy	14 (46.7%)	15 (50%)		
Lobectomy	16 (53.3%)	15 (50%)		
Sufentanil dose during surgery (μg/kg)	0.43 ± 0.11	0.54 ± 0.10	− 4.457	<0.001*
Remifentanil dose during surgery (μg/kg·min)	0.10 ± 0.05	0.12 ± 0.04	0.050	0.960
Duration of anesthesia (min)	142 ± 42	166 ± 56	− 1.772	0.081
Duration of surgery (min)	109 ± 38	131 ± 54	− 1.676	0.099
Extubation time (min)	20 (10, 20.75)	20 (13.75, 30)	− 0.818	0.414
Patients received ≥ two doses of vasopressors (*n* (%))	5 (8.1%)	6 (20%)	6.107	0.411
Resting pain score at 48 h	0 (0, 1.25)	1 (0, 1.25)	− 1.168	0.243
Coughing pain score at 48 h	3 (3, 3)	3 (2, 4)	− 0.775	0.439
Cumulative equivalent sufentanil dose at 48 h (μg/kg)	0.52 ± 0.13	0.69 ± 0.22	− 3.771	0.001*
Chest tube drainage (days)	2.5 (2, 3)	2.5 (2, 3)	− 0.219	0.827
Hospital stay (days)	3.5 (3, 4)	4 (3, 5)	− 0.237	0.813
Incidence of DNR (*n* (%))	9 (30%)	15 (50%)	2.500	0.187
DNR in middle-aged	9/18 (50%)	13/15 (86.7%)		
DNR in low level education	6/10 (60%)	13/15 (86.7%)		
*Preoperative Cogstate test*				
Detection (log10 ms)	2.71 ± 0.13	2.69 ± 0.12	− 1.064	0.292
Identification (log10 ms)	2.88 ± 0.12	2.88 ± 0.13	− 1.249	0.217
*Postoperative Cogstate test*				
Detection (log10 ms)	2.80 ± 0.13	2.83 ± 0.15	− 1.064	0.292
Identification (log10 ms)	2.93 ± 0.13	2.97 ± 0.11	− 1.249	0.217
One-card learning (arcsine accuracy)	0.91 ± 0.09	0.90 ± 0.08	0.173	0.864
One-back memory (arcsine accuracy)	1.17 ± 0.22)	1.10 ± 0.26	1.038	0.305

*Group ESPB* group erector spinae plane block, *Group Control* group intercostal nerve block. Oxycodone 1 mg = sufentanil 1ug (Han et al. 2018). *DNR* delayed neurocognitive recovery. Data are shown as mean ± SD, median (Q1, Q3), or numbers (%). *P < 0.05

Delayed neurocognitive recovery or cognitive decline was defined as 1.96 SD decrease in 2 tests (Evered and Silbert 2018). Four different tasks provided by CCB including “detection task (DET),” “identification task (IDN),” “one card learning task (OCL),” and “one-back task (OBK)” were used to assess psychomotor speed, visual attention, visual memory, and working memory. Psychomotor speed (longer reaction time in DET) and visual attention (longer reaction time in IDN) were the two tests that patients showed cognitive decline.

There were 9 patients in the ESPB group and 15 patients in the control group who developed cognitive decline at 48 h after surgery, the incidence of cognitive decline being equivalent between these two groups (9/30 (30%) vs. 15/30 (50%), *P* = 0.187).

For patients who had delayed neurocognitive recovery, compared to the basic performance before surgery, the postoperative performance on DET (psychomotor speed) and IDN (visual attention) in both groups were significantly declined (*P* < 0.05). The reaction time of DET (2.96 ± 0.07 vs. 2.71 ± 0.07 log 10 ms, *P* = 0.000) and IDN (3.06 ± 0.07 vs. 2.87 ± 0.09 log 10 ms, *P* = 0.000) were significantly prolonged 48 h after surgery (*P* < 0.001).

**Fig. 2:**
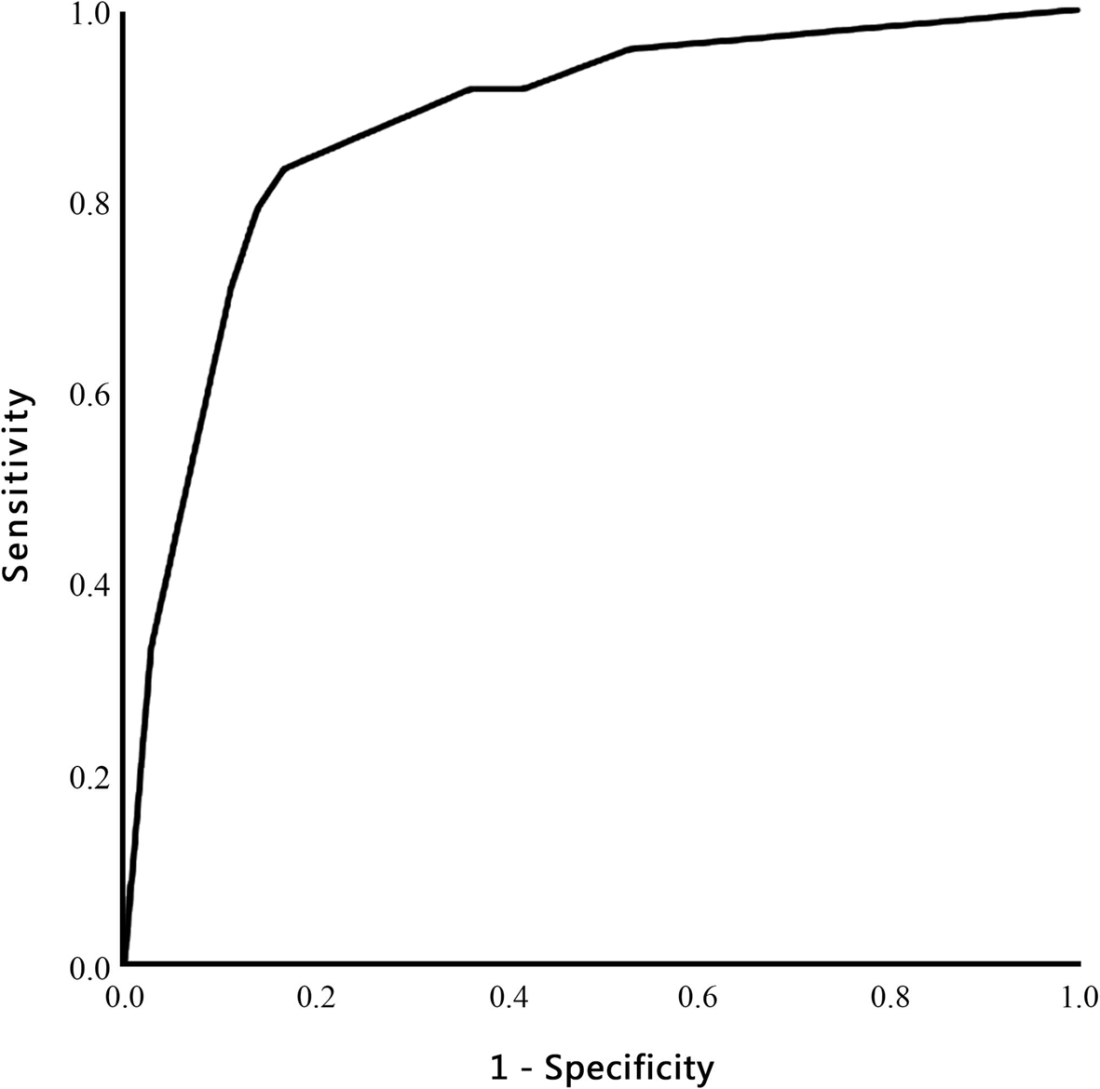
Receiver operating characteristics curves generated to predict the incidence of delayed neurocognitive recovery shortly after video VATS. Area under the curve is 0.879 (95% CI 0.787, 0.971, *P* = 0.031) produced with age > 53.5 years and education years less than 9. VATS video-assisted thoracoscopic surgery

Ever since there was no difference in either demographic or surgical data, all patients were put on logistic regression to identify risk factors for the occurrence of delayed neurocognitive recovery (Table 3). Age, education received less than 9 years, active alcohol drinking, and smoking entered the multivariate regression analysis and yielded a statistically significant regression model (Table 4, Fig. 2). A cut-off value for age was identified as 53.5 years of age using Youden index (OR 9.213 (1.789, 47.437), *P* = 0.008). Meanwhile, low level of education (education years < 9) was also an independent risk factor (OR 5.811, 95% CI 1.419–23.798, *P* = 0.014). The area under the receiver operating curve (ROC) was 0.879, 95% CI 0.787, 0.971, *P* = 0.031.

**Table 3:** Univariate analysis for the development of delayed neurocognitive recovery at post-op 3 day

	Patients with DNR at 3rd day (*n* = 24)	Patients without DNR at 3rd day (*n* = 36)	Statistics	*P* value
Male (*n* (%))	12 (50%)	20 (55.5%)	0.096	0.798
Age (years)	61 ± 8	48 ± 14	3.881	<0.001*
ASA = 2 (*n* (%))	16 (66.7%)	19 (52.8%)	1.396	0.294
Hypertension (*n* (%))	13 (54.1%)	5 (13.9%)	11.566	0.001*
Nerve blocks			2.5	0.187
ESPB group (*n* = 30)	9 (37.5%)	21 (58.3%)		
Control group (*n* = 30)	15 (62.5%)	15 (41.6%)		
Sufentanil dose during surgery (μg/kg)	0.40 ± 0.12	0.35 ± 0.13	1.977	0.05
Remifentanil dose during surgery (μg/kg/min)	0.07 ± 0.04	0.06 ± 0.03	1.324	0.188
Resting pain score at 24h	1 (0, 1)	1 (0, 2)	− 0.364	0.716
Coughing pain score at 24h	3 (2.5, 3)	3 (3, 3)	− 0.339	0.735
Cumulative equivalent sufentanil dose at 48 h (μg/kg)	0.74 ± 0.18	0.66 ± 0.26	0.989	0.330
Chest tube drainage (days)	2 (2, 3)	3 (2, 3.5)	− 0.790	0.429
Hospital stay (days)	3 (3, 4)	4 (3, 5)	− 1.238	0.216
Education years less than 9	18 (75%)	6 (16.7%)	18.929	<0.001*
Smoking (*n* (%))	11 (45.8%)	7 (19.4%)	5.070	0.043*
Drinking (*n* (%))	12 (50%)	7 (19.4%)	6.577	0.022*
*Postoperative Cogstate test*				
Detection (log10 ms)	2.96 ± 0.07	2.71 ± 0.07	12.329	<0.001*
Identification (log10 ms)	3.06 ± 0.07	2.87 ± 0.09	7.817	<0.001*
One-card learning (arcsine accuracy)	0.89 ± 0.09	0.92 ± 0.08	− 2.086	0.204
One-back memory (arcsine accuracy)	1.10 ± 0.20	1.15 ± 0.25	− 0.781	0.439

*DNR* delayed neurocognitive recovery. Data are shown as mean ± SD, median (Q1, Q3), or numbers (%). *P < 0.05

**Table 4:** Risk factors for the development of delayed neurocognitive recovery 48 h after surgery

Age > 53.5 years	*B*	OR	95% CI		*P*
	2.463	9.213	1.789	47.437	0.008*
Education < 9 years	− 2.777	6.829	1.068	43.677	0.042*
Drinking	1.516	4.554	0.974	21.296	0.054

*P < 0.05

For subgroup analysis, there were 18 patients and 15 patients who were more than 53.5 years of age in the ESPB group and the control group respectively. There were 9 patients and 12 patients suffered from delayed neurocognitive recovery in two groups respectively (9/18 (50%) vs. 12/15 (75%), *χ*^2^ =3.182, *P* = 0.074). For patients who received less years of education among those who were more than 53.5 years of age, the presence of delayed neurocognitive recovery was significantly higher in the control group than the ESPB group (11/11 (100%) vs. 6/10 (60%), *P* = 0.035).

## Discussion

This prospective cohort study demonstrated the benefit of preoperative ESPB in reducing perioperative opioid consumption and the occurrence of early cognitive decline, i.e., delayed neurocognitive recovery in high-risk patients. Risk factors identified for delayed neurocognitive recovery during hospitalization after VATS were middle-aged people and less than 9 years of education.

Preoperative ESPB reduced the perioperative opioid consumption through blocking afferent nociceptive stimuli in our study. During general anesthesia, nociceptive signals are continuously generated even though patients have no subjective experiences. Opioids play an important role in perioperative period due to their definite analgesic potency (Shanthanna et al. 2021). Opioid-sparing effect of preoperative nerve block technique conveys benefit in reducing opioid consumption, thus the systematic adverse effects including physical and bowel function (Fleisher 2018). Opioid-sparing analgesic regimen showed benefit in reducing delayed neurocognitive recovery in high-risk patients could be partly explained by the anti-inflammatory effect of nerve blocks in our study.

In our study, delayed neurocognitive recovery affected 30% patients in the ESPB group and 50% in the control group, with an overall incidence of 40% in the study population, which was consistent with previous studies demonstrating 41.4% of elderly patients suffering from delayed neurocognitive recovery after major noncardiac surgery (Monk et al. 2008). However, both middle-aged and elderly people were prone to suffer from cognitive decline compared to younger patients, which was consistent with Hogan’s findings. Hogan demonstrated that people who had surgery between 54 to 58 years of age were susceptible to a decline of memory and verbal learning in comparison with their counterparts who had no surgery, detected by cognitive objective assessment in Wisconsin Registry for Alzheimer’s Prevention cohort (Bratzke et al. 2018). This implicated that for those middle-aged people, who are in the process of early aging, surgery raised additional stress on this process (Patel et al. 2016). As worse ASA status was related with incidence of cognitive decline in Hogan’s study, middle-aged people should have their medical conditions optimized before surgery. Also, those people who developed immediate cognitive decline after surgery should be followed up for mild cognitive impairment or late onset of Alzheimer’s disease in long-term run (De Santi et al. 2008).

Low levels of education contributed to the development of delayed neurocognitive recovery for patients undergoing VATS in our study. The exact pathogenesis for postoperative cognitive decline is unknown, but it is reasonable to attribute this to the combination of surgery, anesthesia, and patients. Cerebral hypoxemia and hypotension was causally related with happening of cognitive decline but patients who did not have hypoxemia or hypotension suffered from cognitive decline also (Moller et al. 1998). Thereafter, “a mine that has been fused and is lying in wait” is well recognized as an explanation, i.e., people who are susceptible to cognitive decline would develop it after trauma, inflammation, or surgery-related stress (Evered et al. 2011). People with low levels of education are with limited cognitive reserve or have cognitive inactivity thus are susceptible to cognitive decline after surgery. Cognitive inactivity is among the seven key modifiable risk factors for the development of cognitive decline, i.e., diabetic mellitus, hypertension, obesity, smoking, depression, cognitive inactivity, and physical inactivity (Deckers et al. 2015). Luckily, several intervention components have been identified, nutrition, physical exercise, cognitive training, management of vascular and metabolic risk factors, and stimulation of social engagement (Ngandu et al. 2015). In our study, all the middle-aged (> 53.5 years) patients, who also did not finish their 9 years of compulsory education, developed early cognitive decline, in contrast to 6 out of 10 patients who received preoperative ESPB. This further strengthened the benefit of implementing enhanced recovery scheme, including which advocates pre-rehabilitation, cognitive training, and application of regional anesthesia.

In our study, when ESPB was applied to high-risk patients, it tended to reduce the incidence of delayed neurocognitive recovery. Well-managed pain could help reduce the occurrence of cognitive decline because postoperative acute or inflammatory pain could exacerbate memory deficits demonstrated through animal studies (Zhang et al. 2013). Surgical trauma and pain induce systemic inflammatory response and release of systemic inflammatory mediators, which enter the central nervous system via the relatively permeable blood-brain barrier and activate the microglia cells to secrete additional cytokines creating a central inflammatory state (Cibelli et al. 2010). It has been reported that 2 biomarkers of neuronal injury, neurofilament light and tau, are increased in plasma after anesthesia and surgery (Evered et al. 2018b). Kristek found that the use of epidural levobupivacaine lowered some inflammatory markers levels and the incidence of delayed neurocognitive recovery compared with intravenous morphine analgesia in the elderly patients undergoing femoral fractures (Kristek et al. 2019). Thus, the anti-inflammatory effect of local anesthetics might contribute to the reduction of cognitive decline in ESPB group (Hollmann and Durieux 2000). Furthermore, a multimodal analgesic regimen involving NSAIDs achieving pain score less than 4 might also convey benefit in cognitive protection after surgery.

We propose two explanations for that ESPB could not reduce the prolongation of detection for patients undergoing VATS under general anesthesia. Firstly, as the analgesic effect of single shot of bi-level ESPB persists 14–16 h, it cannot provide continuous anti-inflammatory effect during the perioperative period. Secondly, the anti-inflammatory effect of continuous NSAIDs applied to both groups helped with preventing delayed neurocognitive recovery.

This study had several limitations. Firstly, the sample size was relatively small to fully demonstrate the influence of the investigated ESPB on the systemic inflammatory response and the consequent appearance of delayed neurocognitive recovery. Secondly, it is an observational study, even though demographic and surgical data did not differ between ESPB and the control group. Thirdly, long-term effect of acute phase cognitive decline after surgery was not investigated. Due to the patients coming from different areas of the country, cognitive assessment was not repeated regularly after surgery. Further studies and long-term follow-up should be established in the future.

In conclusion, preoperative ESPB could reduce perioperative opioid consumption undergoing VATS and tended to lower the appearance of delayed neurocognitive recovery in high-risk patients. Risk factors identified for delayed neurocognitive recovery during hospitalization after VATS were middle age and less than 9 years of education.

## Data Availability

The datasets used and analyzed during the current study are available from the corresponding author on reasonable request.

## References

[CR1] Aldecoa C, Bettelli G, Bilotta F, Sanders RD, Audisio R, Borozdina A (2017). European Society of Anaesthesiology evidence-based and consensus-based guideline on postoperative delirium. Eur J Anaesthesiol.

[CR2] Awada HN, Luna IE, Kehlet H, Wede HR, Hoevsgaard SJ, Aasvang EK (2019). Postoperative cognitive dysfunction is rare after fast-track hip- and knee arthroplasty - but potentially related to opioid use. J Clin Anesth.

[CR3] Bratzke LC, Koscik RL, Schenning KJ, Clark LR, Sager MA, Johnson SC (2018). Cognitive decline in the middle-aged after surgery and anaesthesia: results from the Wisconsin Registry for Alzheimer's Prevention cohort. Anaesthesia..

[CR4] Cibelli M, Fidalgo AR, Terrando N, Ma D, Monaco C, Feldmann M (2010). Role of interleukin-1beta in postoperative cognitive dysfunction. Ann Neurol.

[CR5] De Santi S, Pirraglia E, Barr W, Babb J, Williams S, Rogers K (2008). Robust and conventional neuropsychological norms: diagnosis and prediction of age-related cognitive decline. Neuropsychology..

[CR6] Deckers K, van Boxtel MP, Schiepers OJ, de Vugt M, Munoz Sanchez JL, Anstey KJ (2015). Target risk factors for dementia prevention: a systematic review and Delphi consensus study on the evidence from observational studies. Int J Geriatr Psychiatry.

[CR7] Evered LA, Silbert BS (2018). Postoperative cognitive dysfunction and noncardiac surgery. Anesth Analg.

[CR8] Evered LA, Silbert BS, Scott DA, Maruff P, Ames D, Choong PF (2011). Preexisting cognitive impairment and mild cognitive impairment in subjects presenting for total hip joint replacement. Anesthesiology..

[CR9] Evered L, Silbert B, Scott DA, Ames D, Maruff P, Blennow K (2016). Cerebrospinal fluid biomarker for alzheimer disease predicts postoperative cognitive dysfunction. Anesthesiology..

[CR10] Evered L, Silbert B, Knopman DS, Scott DA, DeKosky ST, Rasmussen LS (2018). Recommendations for the nomenclature of cognitive change associated with anaesthesia and surgery-2018. Anesthesiology..

[CR11] Evered L, Silbert B, Scott DA, Zetterberg H, Blennow K (2018). Association of changes in plasma neurofilament light and tau levels with anesthesia and surgery: results from the CAPACITY and ARCADIAN studies. JAMA Neurol.

[CR12] Fleisher LA (2018). Regional anesthesia: what we need to know in the era of enhanced recovery after surgery protocols and the opioid epidemic. Anesthesiol Clin.

[CR13] Han L, Su Y, Xiong H, Niu X, Dang S, Du K (2018). Oxycodone versus sufentanil in adult patient-controlled intravenous analgesia after abdominal surgery: a prospective, randomized, double-blinded, multiple-center clinical trial. Medicine (Baltimore).

[CR14] Hollmann MW, Durieux ME (2000). Local anesthetics and the inflammatory response: a new therapeutic indication?. Anesthesiology..

[CR15] Kristek G, Rados I, Kristek D, Kapural L, Neskovic N, Skiljic S (2019). Influence of postoperative analgesia on systemic inflammatory response and postoperative cognitive dysfunction after femoral fractures surgery: a randomized controlled trial. Reg Anesth Pain Med.

[CR16] Moller JT, Cluitmans P, Rasmussen LS, Houx P, Rasmussen H, Canet J (1998). Long-term postoperative cognitive dysfunction in the elderly ISPOCD1 study. ISPOCD investigators. International Study of Post-Operative Cognitive Dysfunction. Lancet..

[CR17] Monk TG, Weldon BC, Garvan CW, Dede DE, van der Aa MT, Heilman KM (2008). Predictors of cognitive dysfunction after major noncardiac surgery. Anesthesiology..

[CR18] Ngandu T, Lehtisalo J, Solomon A, Levalahti E, Ahtiluoto S, Antikainen R (2015). A 2 year multidomain intervention of diet, exercise, cognitive training, and vascular risk monitoring versus control to prevent cognitive decline in at-risk elderly people (FINGER): a randomised controlled trial. Lancet..

[CR19] Patel D, Lunn AD, Smith AD, Lehmann DJ, Dorrington KL (2016). Cognitive decline in the elderly after surgery and anaesthesia: results from the Oxford Project to Investigate Memory and Ageing (OPTIMA) cohort. Anaesthesia..

[CR20] Shanthanna H, Ladha KS, Kehlet H, Joshi GP (2021). Perioperative opioid administration. Anesthesiology..

[CR21] Zhang X, Xin X, Dong Y, Zhang Y, Yu B, Mao J (2013). Surgical incision-induced nociception causes cognitive impairment and reduction in synaptic NMDA receptor 2B in mice. J Neurosci.

[CR22] Zhao H, Xin L, Feng Y (2020). The effect of preoperative erector spinae plane vs. paravertebral blocks on patient-controlled oxycodone consumption after video-assisted thoracic surgery: a prospective randomized, blinded, non-inferiority study. J Clin Anesth.

